# The chick chorioallantoic membrane as an *in vivo* xenograft model for Burkitt lymphoma

**DOI:** 10.1186/1471-2407-14-339

**Published:** 2014-05-18

**Authors:** Marcel Klingenberg, Jürgen Becker, Sonja Eberth, Dieter Kube, Jörg Wilting

**Affiliations:** 1 Department of Anatomy and Cell Biology, University Medical Center Goettingen, Kreuzbergring 36, Goettingen 37075, Germany; 2 Department of Hematology and Oncology, University Medical Center Goettingen, Robert-Koch-Strasse 40, Goettingen 37075, Germany

**Keywords:** Non-Hodgkin lymphoma, Angiogenesis, Lymphogenic metastasis, BL2, BL2B95, Tumor-stroma interaction, Microenvironment, Macrophages, Granulocytes

## Abstract

**Background:**

Burkitt lymphoma (BL) is an aggressive malignancy that arises from B-cells and belongs to the group of Non-Hodgkin lymphomas (NHL). Due to the lack of appropriate *in vivo* models NHL research is mainly performed *in vitro*. Here, we studied the use of the chick chorioallantoic membrane (CAM) for the generation of human BL xenograft tumors, which we compared with known characteristics of the human disease.

**Methods:**

In order to generate experimental BL tumors, we inoculated human BL2B95 and BL2-GFP cells on the CAM. BL2B95 xenograft-tumors were grown for seven days and subsequently analyzed with transmission electron and immunofluorescence microscopy, as well as histological staining approaches. BL2-GFP cells were studied at regular intervals up to seven days, and their metastatic behavior was visualized with intravital immunofluorescence techniques.

**Results:**

Xenografted BL2B95 cells formed solid tumors in the CAM model with a Ki67-index greater than 90%, preservation of typical tumor markers (CD10, CD19, CD20), a ‘starry sky’ morphology, production of agyrophilic fibers in the stroma, formation of blood and lymphatic vessels and lymphogenic dissemination of BL2B95 to distant sites. We identified macrophages, lymphocytes and heterophilic granulocytes (chick homolog of neutrophils) as the most abundant immune cells in the experimental tumors. BL2-GFP cells could be traced in real-time during their distribution in the CAM, and the first signs for their dissemination were visible after 2-3 days.

**Conclusions:**

We show that xenografted BL2B95 cells generate tumors in the CAM with a high degree of cellular, molecular and proliferative concord with the human disease, supporting the application of the CAM model for NHL research with a focus on tumor-stroma interactions. Additionally we report that BL2-GFP cells, grafted on the CAM of ex ovo cultured chick embryos, provide a powerful tool to study lymphogenic dissemination in real-time.

## Background

The term Non-Hodgkin-lymphoma (NHL) describes all malignant diseases of the lymphatic system not belonging to the class of Morbus Hodgkin. The distinction between Morbus Hodgkin and NHL is based on the occurrence of multinucleated Sternberg-Reed cells. When these are detected in microscopical examinations, the malignancy is classified as Morbus Hodgkin lymphoma. Various factors can lead to the formation of NHL, including chromosomal translocations and viral infections. NHL can be further subdivided according to the affected cell type into B- and T-cell lymphomas. In this study we analyzed the NHL subtype Burkitt lymphoma (BL), which arises from B-cells. BL is a very rare, aggressive disease with an incidence rate of approximately 0.2 per 100.000/year [1]. The malignancy is divided into three subgroups and often associated with the Epstein-Barr-Virus (EBV), which drives transformation [2]. BL was first described by Denis Burkitt in 1958, and this subtype is nowadays considered as the endemic, equatorial African form of the disease [3]. In this study we used the well established BL2 cell line, and a derivative of this, which was *in vitro* infected with EBV serotype B95-8 [4]. The BL2 cell line was initially isolated from a Caucasian patient with multiple metastases, which involved the central nervous system and the bone marrow. The cell line therefore represents a sporadic but aggressive subtype of BL [5].

Although there is a good chance of cure for NHL patients treated with stringent chemotherapeutic regimens, there are a small percentage of cases that are resistant to therapy [6,7]. These patients cannot be identified by studies of isolated tumor cells, and it is assumed that there are specific tumor-stroma interactions that render lymphoma cells resistant to chemotherapy. There are mouse models to study the interactions of lymphomas with their microenvironment [8], but it appears unlikely that mouse models can be performed in sufficiently high quantities that allow global systems-biological analyses of tumor-stroma interactions with and without divers chemotherapeutic regimens. We have recently shown that BL cell lines can successfully be inoculated on the chick chorioallantoic membrane (CAM) [9,10]. Several CAM experiments described in detail the tumor microenvironment and the metastatic dissemination of various tumor entities including melanoma, glioma, fibrosarcoma and colon carcinoma [11-13]. This underlines the upcoming role of the CAM model in cancer research, especially in the field of tumor-stroma interactions and the analysis of the metastatic cascade. Experiments based of the CAM-tumor model appear to be highly suited to study the aforementioned aspects due to the fact that the CAM provides the presence of nearly all relevant stroma factors, e.g. immune cells, extracellular matrix components, blood and lymphatic vessels.

The formation of the CAM starts around day 4 of chick embryo development. It is an extra-embryonic organ, which develops by the fusion of the chorion with the vascularized allantoic membrane. The CAM is responsible for the gas exchange of the embryo and for that reason very well perfused. It shows a high density of blood and lymphatic vessels, which explains its main usage as a model for angiogenesis [14]. In addition, the CAM is also established as an animal model for cancer research [9,11,15]. However, very few studies have dealt with the CAM in the context of hematological malignancies [16-18], and, to the best of our knowledge, there are no studies characterizing the host leukocytes that infiltrate the grafted tumors. Previous experiments in our lab showed that the BL cell line, BL2B95, develops tumors in the CAM that exhibit high similarities to human BL [9,10]. Here, we followed up on these studies and validated the usefulness of the CAM model for lymphoma research. We show a high degree of molecular and morphological concord, including tumor-stoma interactions, with the human disease, supporting the application of the CAM as an *in vivo* model for NHL research.

## Methods

### Cell culture

The Burkitt lymphoma cell lines BL2B95 were cultured in BL-medium (RPMI 1640 medium with 10% FCS, 1% penicillin/streptomycin, 10 mM HEPES, 1 mM sodium-pyruvate, 50 μM α-thioglycerol and 20 nM BCS). Cells were cultured in cell culture flasks and incubated at 37°C and 5% CO_2_. BL2-GFP (BL-2 ns-c* GFP) cells were cultured in RPMI 1640 with 10% FCS and 1% penicillin/streptomycin.

### Stable transduction of GFP

A self-inactivating lentivirus was prepared by transient transfection of 293 T cells using calcium phosphate precipitation method. Briefly, pGIPZ ns-control (Thermo Scientific, Schwerte, Germany) encoding GFP and a non-silencing control shRNA (ns-c) was co-transfected with packaging vector pCMVΔr8.91 and envelope vector pVSV-G in a ratio of 3:2:1 into 293 T cells. After harvesting and determination of titer, lentivirus supernatant was added to BL2 cells at a MOI < 1 in the presence of 10 μg/ml protamine sulfate, and samples were centrifuged for 1.5 h at 850 g and 37°C. After 2 days 1 μg/ml puromycin was added to select stably transduced cells expressing GFP. The GFP expression in puromycin-resistant cells was analyzed with a flow cytometer and, when the cells were positive, they were expanded (Additional file 1: Figure S1).

### CAM assay with BL2B95 cells

Fertilized White Leghorn chick eggs were incubated at 80% relative humidity and 37.8°C. The eggs were windowed at day 3 and the window was sealed with cellotape. At day 10 of chick development, one million BL2B95 cells/egg were applied on the CAM. Cells were resuspended in 50% BL-medium and 50% Matrigel and incubated for 30 min at 37°C, 5% CO_2_ before applying them on the CAM. The tumors were dissected on day 17 of chick development. Tumors were fixed in 4% paraformaldehyde for 15 min, washed thrice in PBS and transferred into 10% sucrose for 3 h at 4°C and 30% sucrose overnight at 4°C. Tumors were then embedded in tissue freezing medium and cut with a cryotome into 4-12 μm thick sections. The experiments were performed according to the guidelines of the European Parliament (2010/63/EU) and the council for the protection of animals in science (§14 TierSchVersV).

### CAM assay (*ex ovo*) with intravital imaging of BL2-GFP cells

Specific pathogen free fertilized White Leghorn chick eggs were incubated for 72 h at >80% relative humidity and 37.8°C. On developmental day 3 the eggs were cracked open and the embryo was carefully transferred into a plastic square weighing boat (89×89×25mm) and cultured until day 17 of embryonic development (Additional file 2: Figure S2 A-G). The weighing boat was placed in a tissue culture flask with a re-closable lid (Additional file 2: Figure S2 H). 13 ml of purified water (0.1% copper sulfate) were added to ensure sufficient humidity. On day 10, 10^6^ BL2-GFP cells in varying percentages of Matrigel (10-50%) or without Matrigel were inoculated on the CAM (Additional file 3: Figure S3). The embryos were incubated until day 17 in the above mentioned conditions (>80% rh, 37.8°C). Pictures were taken every 24 h with Leica MZ16FA microscope. Procedures were adopted from [13]. The experiments were performed according to the guidelines of the European Parliament (2010/63/EU) and the council for the protection of animals in science (§14 TierSchVersV).

### Histological staining

HE, panoptic Pappenheim, Trichrome and Gomori silver staining were performed according to standard procedures [19].

### Transmission electron microscopy

Specimens with an approximate volume of 600 mm^3^ were fixed with Karnovsky fixative for at least two hours, washed in 0.15 M phosphate buffer for 10 min, transferred into osmium tetroxide solution and incubated for 2 h at 4°C. Then the samples were rinsed with 0.15_M phosphate buffer for 10 min and subsequently dehydrated in an ascending ethanol series of 30%, 50%, 70%, 90% and two times absolute ethanol for 10 min each. Next the samples were incubated twice in 100% propylene oxide for 10 min at 4°C. They were then incubated for 1 h at 4°C in 50% propylene oxide and 50% glycid ether, transferred into 25% propylene oxide and 75% glycid ether and incubated over night at 4°C. Then the samples were embedded in epon embedding solution and incubated for 24 h at 60°C. The embedded tissue was cut with an Ultracut E microtome (Reichert-Jung) to 90 nm sections and transferred onto formvar-coated grids. After air-drying samples were incubated 10 min in 1% uranyl acetate solution, 10 min in lead citrate (Reynolds) and rinsed with purified water. Specimens were analyzed with a Leo 906E (Zeiss) transmission electron microscope.

### Immunofluorescence analyses

Immunofluorescence staining of specimens was performed by incubation for 1 h with blocking reagent (PBS, 1% BSA, 5% goat serum, 0.2% Triton X-100), 1 h incubation of primary antibody diluted in antibody solution (TBS [0.05 M, pH 7.2-7.4], 1% BSA, 0.5% Triton X-100) and 1 h incubation of secondary antibody diluted in antibody solution mixed with DAPI (1:10,000). After every step specimens were rinsed thrice with PBS. Samples were mounted with Fluoromount-G (Sigma-Aldrich) and dried over night at room temperature. Stained specimens were studied with Zeiss Axio Imager.Z1 (Carl Zeiss Goettingen) and filter sets 38HE, 43, 49 and 50. Primary antibodies were rabbit-anti-human Prox1 (Relia Tech) at a 1:500 dilution, mouse anti-human HLA A,B,C (BioLegend) at 1:200 dilution and mouse anti-Mep21 (chick CD34 homolog; M. Williams, AbLab) at dilution of 1:100. Secondary antibodies (Invitrogen) were Alexa Fluor® 594 goat anti-mouse IgG (H + L), Alexa Fluor® 488 goat anti-rabbit IgG (H + L), Alexa Fluor® 660 goat anti-rabbit IgG (H + L), highly cross-adsorbed; Alexa Fluor® 594 goat anti-mouse IgG2a (γ2a); Alexa Fluor® 488 goat anti-mouse IgG1 (γ1); at a dilution of 1:200 in antibody solution.

### Immunohistolochemical analyses

Cryosections were fixed in 100% methanol for 3 min, incubated for 3 min in TBS/0.1% Tween, and transferred into 3% H_2_O_2_. Specimens were then washed thrice in TBS/0.1% Tween, blocked with PBS/1% BSA. Subsequently anti-Ki67 antibody was added (rabbit mAb, clone D3B5, Cell Signaling Technology, Danvers, MA, USA) at a concentration of 1:200 (diluted in PBS/1% BSA) and incubated over night at 4°C on a rocking table. Specimen were then washed thrice with TBS/0.1% Tween, secondary HRP-conjugated goat-anti-rabbit antibody (St. Cruz Biotechnology, Heidelberg, Germany) was added at a concentration of 1:200 (diluted in PBS/1%BSA) and incubated for 30 min at room temperature. After that the sections were washed thrice with TBS/0.1% Tween, incubated for 5 min in TrisHCl-buffer/0.125% ammonium sulfate/0.05% DAB/0.015% H_2_O_2_ and washed with tab water. Section were then counterstained with 0.1% nuclear fast red-aluminum sulfate solution (Merck Millipore, Darmstadt, Germany), washed with tab water, incubated twice in 100% ethanol for 3 min, and incubated twice in xylene for 3 min. Samples were mounted with DePeX (Serva, Heidelberg, Germany). Staining with antibodies against CD20, CD19, CD10, CD5, TdT (IR604, IR656, IR648, IR082, IR001; Dako, Hamburg, Germany), and HLA A,B,C (BioLegend) was performed as described above except for the counterstaining, which was performed with hematoxylin, but omitted for CD10 and HLA.

### Western blot

Immunoblot analyses were performed as described previously [20]. Bcl-6 antibody was obtained from Cell Signaling, c-Myc antibody was from Abcam, alpha-Tubulin antibody was from Millipore.

### ^3^H thymidine assay

Proliferation of cell lines was assessed using ^3^H thymidine incorporation assay as recently described [10].

## Results

### Tumor formation

**Figure 1:**
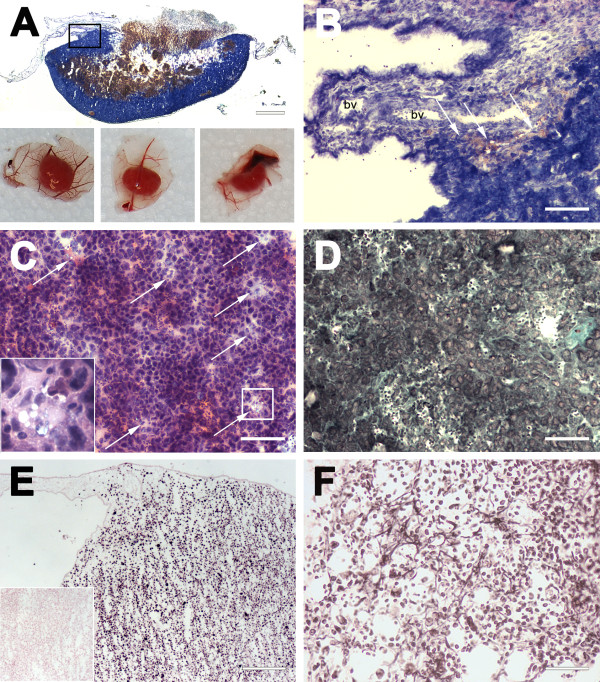
**Histological staining of BL2B95 tumors on CAM. A** (upper part): Panoptic Pappenheim staining of tumor cryosections. The tumor can be divided into a dark stained zone and a central necrotic/hemorrhagic area surrounded by the non-infiltrated CAM. Scale bar = 1 mm. Lower part: Photopgraphs of BL2B95 tumors. **B**: Panoptic Pappenheim staining of tumor/CAM border. BL2B95 cells show dark basophilic cytoplasm. CAM appears grayish blue with two blood vessels (bv) in the center of the picture. The tumor periphery is infiltrated by chick leukocytes, which are clearly visible due to their numerous reddish granules (arrows). Scale bar = 200 μm **C**: HE staining of tumors shows classical ‘starry sky’ appearance of BLs with tingible body macrophages (arrows and insert). Scale bar = 100 μm. **D**: Trichrome staining shows the small connective tissue proportion (green) of BL2B95 tumors. Scale bar = 100 μm. **E**: Ki67 immunohistochemical staining. More than 90% of the tumor cells are Ki67^+^. Insert shows negative control. Scale bar = 200 μm. **F**: Gomori silver staining of a tumor area with massive chick leukocyte infiltration; black fibres indicate agyrophilic fibres. Scale bar = 100 μm.

BL2B95 were inoculated on the CAM of day-10 chick embryo development. The embryos were sacrificed after 7 days and BL2B95-derived tumors were dissected. We observed that BL2B95 cells formed solid tumors on the CAM in 100% of the experiments (n = 63). Tumors formed by BL2B95 assumed a lentiform shape and varied in color between reddish (highly vascularized) and whitish (sparsely vascularized) (Figure 1A). BL2-GFP cells formed tumors on the CAM, too, but showed uniformly a more whitish color (n = 11), suggesting a higher angiogenic potential of the EBV-transduced cells.

### Histological characteristics of experimental BL-tumors

**Figure 2:**
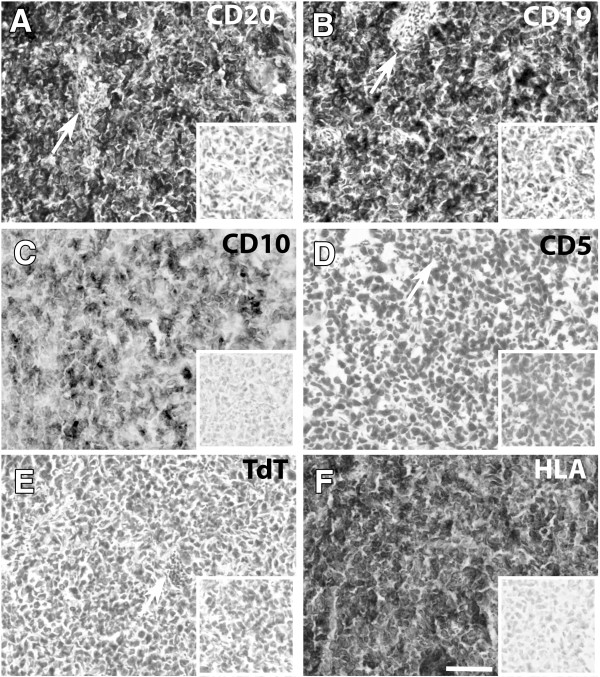
**Immunohistochemical staining on BL2B95 tumors on the CAM. A-F**: Immunohistochemical staining of cryosections (8 μm) of BL2B95 tumors 7 days after inoculation on the CAM, counterstained with hematoxylin (C and F were not counterstained). Inserts in the lower right corner show negative controls. White arrows indicate blood vessels. **A**: CD20, **B**: CD19, **C**: CD10, **D**: CD5, **E**: TdT, **F**: HLA A,B,C. Scale bar = 80 μm. The tumors are positive for CD20, CD19, CD10 and HLA A,B,C, but negative for CD5 and TdT.

Tumor cryosections were analyzed with classical histological staining protocols including HE, panoptic Pappenheim, Trichrome and Gomori silver staining. A modified protocol of the panoptic Pappenheim stain illustrated the immigration of chick leukocytes into the tumor periphery (Figure 1A, B). Thereby, chick macrophages possessed a light-blue cytoplasm and reddish granules, whereas the BL2B95 cells stained dark-blue (basophilic) and were considerably larger than the chick leukocytes. We observed that the borders of the tumors were seamed by immigrating chick macrophages. They were discernible as lightly stained cell clusters in the dark-blue BL2B95 tumor mass. The hematoxylin and eosin staining, too, revealed a ‘starry sky’ appearance of the tumor, consisting of dark and lightly stained areas (Figure 1C). The chick macrophages were visible as light cells loaded with cellular debris and apoptotic tumor cells. This ‘starry sky’-like appearance does reflect the characteristics of human BL histology and shows that, like the primary tumors, the experimental tumors largely consist of BL2B95 cells and interacting leukocytes. Trichrome staining of the tumor specimens showed that they contained small portions of connective tissue, which, upon further analyses by silver staining, was characterized by agyrophilic fibers (Figure 1D, F). These fibers bind Ag^+^-ions during the staining procedure. Agyrophilia is a feature of type-III collagen, which is characteristic of lymphatic tissues and areas of active inflammatory reactions. Ki67 staining of the experimental tumors showed a mitotic index greater than 90% (Figure 1E). The immunohistochemical staining with established BL markers showed that the tumor cells show the characteristic pattern of classical BL. The experimental BL2B95 tumors were positive for CD20, CD19, and CD10 (Figure 2A-C). CD5 and TdT staining turned out to be negative (Figure 2D, E). HLA, which was used to differentiate between human and chick cells, clearly stained all BL cells (Figure 2F).

**Figure 3:**
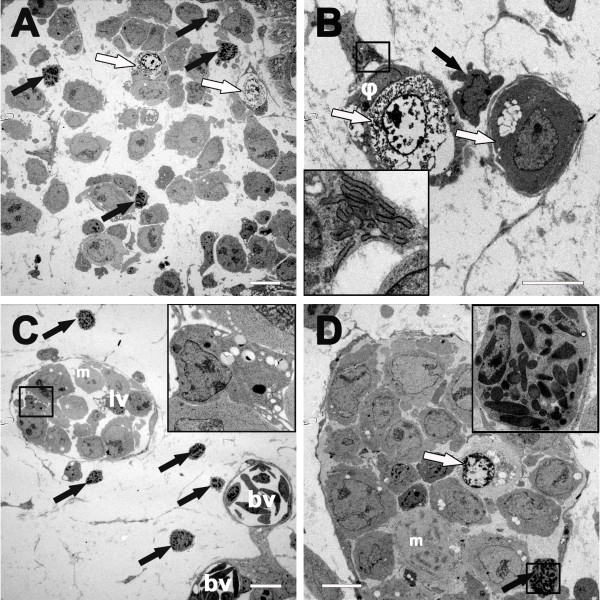
**Transmission electron microscopy of BL2B95 tumors on the CAM. A**: BL2B95 cells applied on the CAM form tumors infiltrated by granulocytes (black arrows). Apoptotic cells are found in the tumors (white arrows). **B**: Some tumor cells are phagocytized by macrophages (φ). Tumor cells are completely surrounded by pseudopodia and undergo apoptosis (left white arrow shows late stage apoptotic cell; right white arrow shows tumor cell in early apoptosis with cytosolic vacuolization). Black arrow shows a lymphocyte in close proximity to the macrophages. Insert shows higher magnification of Birbeck granules, which identify the cell in the rectangle as a dendritic cell. **C**: CAM stroma with lymphatic (lv) and blood vessels (bv) in the tumor periphery. The lumen of the lymphatic vessel is completely filled with BL2B95 cells and chick leukocytes; black arrows indicate granulocytes. The black rectangle indicates the area magnified in the insert, showing a macrophage with phagosomes of different maturation stages. **D**: Lymphatic vessel in the tumor center. The white arrow shows an apoptotic cell. The black arrow shows a granulocyte, which is magnified in the insert. The nucleus of the granulocyte is bi-lobed and the cytoplasm filled with rod-shaped granules. This characterizes the cell as a heterophilic granulocyte, which form the most abundant subgroup of granulocytes in the experimental tumors. (m = mitotically active cell, precondensed chromosomes). Scale bars **(A-D)** = 10 μm.

In order to characterize in greater detail the chick leukocytes, which immigrated into the BL2B95 tumors, we performed transmission electron microscopy (TEM)-based analyses (Figure 3A-D). The studies confirm that the tumors consisted mainly of BL2B95 cells and chick leukocytes (Figure 3A). BL2B95 cell nuclei often presented condensed or precondensed chromosomes, revealing high mitotic activity (Figure 3C, D). The nuclei of the tumor cells were mainly euchromatic with one or several prominent nucleoli. The cytoplasm possessed a lower electron density compared to the chick leukocytes and appeared therefore lighter in the TEM pictures. Lipid vesicles in the BL2B95 cell cytoplasm and plasmalemmal microvesicles were also observed, showing that the BL cells maintained their initial morphologic features in the CAM model (Figure 3A, C, D). The identification of chick leukocyte subgroups was performed according to morphological criteria and revealed the presence of dendritic cells, macrophages and heterophilic granulocytes (chick granulocytes corresponding to human neutrophils) within or in close proximity to the BL2B95 tumors (Figure 3A-D). In accordance with previous studies, lymphogenic dissemination of BL2B95 cells was also observed with this method [9,10]. Thereby the tumor cells often filled the lumen of lymphatic vessels completely (Figure 3C, D).

### Lymphatic dissemination of BL cells

**Figure 4:**
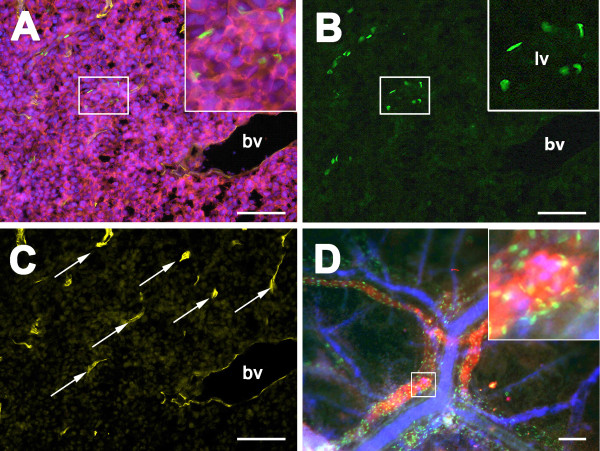
**Immunofluorescence staining of BL2B95 tumors on CAM. A**: Immunofluorescence staining of tumor cryosections, merged picture of four channels. Cells were stained with DAPI (blue), anti-Prox1 (green), anti-HLA A,B,C (red), and anti-MEP21 (yellow). Prox1 stains nuclei of lymphatic endothelial cells, HLA A,B,C probes BL2B95 cells, and MEP21 stains blood vessels (bv). **B**: Green channel of A showing anti-Prox1 staining of nuclei of LECs. **C**: Yellow channel of A showing anti-MEP21 staining. Capillaries (arrows) and larger blood vessels (bv) are visible. **D**: Immunofluorescence staining of a whole mount specimen showing a region approximately 1 cm apart from the solid tumor. Merged picture of three channels. The sample was stained with DAPI (blue), anti-Prox1 (LECs, green), and anti-HLA A,B,C (tumor cells, red). Blood vessels appear as blue lines due to nucleated chick erythrocytes. Note specific localization of tumor cells in lymphatics. Insert: Higher magnification of boxed area. Scale bars **(A-C)** = 100 μm; Scale bar **(D)** = 100 μm.

**Figure 5:**
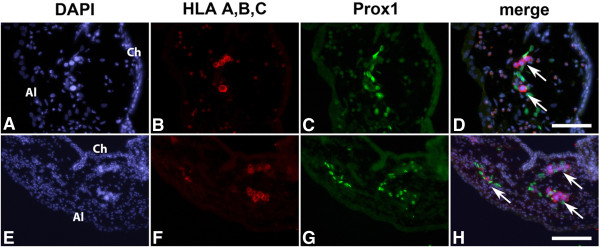
**Immunofluorescence staining of metastatic foci in experimental BL2B95 tumors. A-H**: Immunofluorescence staining of cryosections from experimental BL2B95 tumors. Pictures show BL2B95 cells in lymphatic vessels of the CAM. Cells were stained with DAPI (blue in **A, E**), anti-HLA A,B,C (red in **B, F**) and anti-Prox1 (green in **C, G**). **A, E**: BL2B95 cells can be clearly discriminated due to their large nuclei. Al, allantoic epithelium. Ch, chorionic epithelium. **B, F**: HLA A,B,C staining of BL2B95 cells identifies human cells in the chick stroma. **C, G**: Prox1 staining of chick lymphatic endothelial cells. **D, H**: Merged pictures. Arrows show BL2B95 cells in CAM lymphatics. Scale bars = 50 μm in **D,** and 100 μm in **H**.

**Figure 6:**
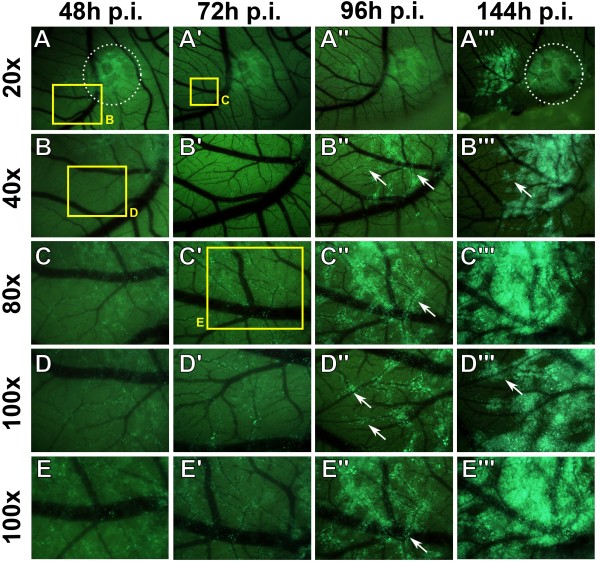
**Intravital imaging of BL2-GFP cells in the *ex ovo *CAM model. A-E**”: Time lapse images of BL2-GFP cells, which were grafted in 10% Matrigel on the *ex ovo* CAM. BL2-GFP cells show a bright green fluorescence, CAM tissue a weak greenish autofluorescence, and blood vessels appear black. Pictures were taken every 24 h, beginning 48 h post inoculation (p.i.). The magnification of the pictures is indicated. The yellow rectangles mark regions shown at higher magnification in **B-E**. The white dotted line in **A** and **A”** depicts the region of initial inoculation. Note the dissemination of tumor cells along vascular routes (white arrows).

The lymphatic dissemination of BL2B95 cells was analyzed by immunofluorescence staining of tumor cryosections and whole-mount specimens (Figure 4A-D). Tumor cells were visualized with anti-HLA-antibodies and lymphatic vessels with lymphatic endothelial cell (LEC)-specific anti-Prox1 antibodies (staining of LEC nuclei). The borders of the tumors appeared fuzzy and several lymphoma cells migrated into the stroma of the CAM, confirming our previous observations [9]. Our analyses of tumor sections showed that BL2B95 cells infiltrated lymphatic vessels both within the tumors (Figure 4A) and in the vicinity of the tumors. Additionally, whole-mount and cryosection staining showed that BL2B95 cells migrated long distances and were found in lymphatic vessels several millimeters apart from the primary tumors (Figure 3C, D; Figure 4D; Figure 5). The dissemination along lymphatics was even more clearly shown for the BL2-GFP cell line (Figure 6; Additional file 3: Figure S3). BL2-GFP cells were visualized with intravital GFP imaging. Pictures were taken every 24 h, beginning 48 h after the inoculation (day 12 of embryonic development). The time lapse image arrangement demonstrates the migratory routes of BL2-GFP cells along the outside of the CAM blood vessels (Figure 6; Additional file 3: Figure S3). The cells were often arranged in a characteristic pattern along the blood vessels, which overlays perfectly with the localization of the lymphatic vessels within the CAM. Immunofluorescence staining of the CAM revealed that blood vessels are often flanked by lymphatic vessels on each side (Figure 4D; Additional file 4: Figure S4). Of note, this technique allows to study the distribution of single GFP^+^ lymphoma cells within the CAM tissue in real-time. The CAM is lucent enough to trace even cells, which have migrated into deeper layers of the CAM, provided that the cells show a strong GFP expression. Thereby, the density of the Matrigel, which was used for the inoculation, was found to influence the timing of the immigration into the CAM stroma and lymphatics. BL2-GFP cells inoculated in 50% Matrigel showed much lesser infiltration of lymphatics compared to cells, which were inoculated with 10% and 0% Matrigel (Table 1). This is most probably caused by the matrix-metalloprotease (MMP)-independent (amoeboid) migratory mode of lymphoma cell lines, which impedes migration through a collagen-rich matrix.

**Table 1:** **Formation of metastatic foci (MF) depend**e**nt on the proportion of matrigel, used in the inoculation process**

**Percent matrigel**	**Mean No. of MF**	**Variance**	**n**
50	0.25	0-1	4
10	2.3	1-5	3
0	0.25	0-1	4

### Lymphatic and blood vessel formation

The determination of the lymphatic and blood vessels was performed by immuno-fluorescence (IF) on experimental BL2B95 tumors. Blood vessels were detected with MEP21 (CD34 homolog) antibody staining, which is a marker for chick blood endothelial cells (BECs). The lymphatic vessels were immunostained in cryosections with anti-Prox1 antibodies, which mark the nuclei of LECs. The IF analyses revealed prominent MEP21^+^ and Prox1^+^ vessels in all BL2B95 tumors (Figure 4B, C). This clearly shows an interaction of both types of vessels with the BL2B95-derived tumors and indicates the secretion of hem- and lymphangiogenic growth factors by the tumors. The presence of blood vessels within the BL2B95 tumors was also observed in the histological stainings, shown in Figure 2. In contrast, BL2-GFP derived tumors showed a lesser degree of vascularization and appeared therefore more whitish.

## Discussion

Our investigations of BL cells in the chicken CAM model show a great degree of conformity with the human disease. BL cells formed solid tumors and disseminated in the animal mainly via the ECM and the lymphatics to distant sites. This is the same behavior as in the human and underlines that the CAM is a highly suited model to investigate the initial steps of BL-stroma interactions and the metastatic behavior even of single tumor cells.

Tumor interactions with various cellular components in the CAM are highly reminiscent of human BL. HE staining showed that the BL2B95 cells form tumors with a ‘starry sky’-like appearance, caused by ‘tingible body macrophages’, which are scattered in the tumors at regular intervals. This is extremely significant because the ‘starry sky’ is the major histological characteristic of BL in humans [21]. Furthermore we were able to identify the involvement of various chick leukocytes in the BL2B95 tumor formation. Transmission electron microscopy (TEM) depicted the presence of heterophilic granulocytes, the avian counterparts of mammalian neutrophils, which, besides macrophages, are the most abundant leukocytes in the tumors. This is in concord with findings of previous studies performed in the CAM [22]. Additionally we found dendritic cells in the tumor, which formed cell-cell contacts with other avian leukocytes, indicating an active immune response of the host. Altogether our findings depict a highly complex tumor-stroma interaction in the CAM model, which can at least partly simulate the situation in the human disease [23].

The formation of blood vessels and lymphatics in the BL2B95 tumors is an additional characteristic, which underlines the highly complex tumor microenvironment in this model. Although we found a varying degree of angiogenesis in the EBV-transduced BL2B95 tumors, the degree of vascularization appeared to be higher than in the EBV-negative BL2 tumors. It is likely that the virus modulates the composition and quantity of immigrating leukocytes, which then secrete angiogenic and lymphangiogenic growth factors. The production of such factors by neutrophils and macrophages has frequently been shown [24]. The variability of the vessel density in tumors derived from the same cell line makes quantitative assessments more laborious than in genetically homogenous, inbred, mice; but this probably better reflects the intra- and inter-individual heterogeneity of human tumors. Although the CAM is an embryonic organ and the immune system of the host is in the process of development, the main components of the immune system are present, which may render the model superior to immunocompromised mouse models.

Despite the species barrier and the embryonic environment, human BL cell lines acquire many of the morphological characteristics, and retain the molecular characteristics, of primary lymphomas when grafted on the chicken CAM. Besides the typical starry sky morphology, the stroma turns into the typical stroma of lymphoid organs, with production of agyrophilic fibers. The morphological features of the BL cells, such as the production of microvesicles, remains unaltered. The B-lymphocyte antigens CD19 and CD20, as well as neprilysin (CD10), which is characteristic of early B-cells, are positive in the CAM lymphomas. The proliferation index of the experimental tumors is greater than 90% (Ki67), which is again a major feature of human BL [25]. The development and maintenance of so many lymphoma characteristics substantiates the comparability of the CAM-tumor model with the human disease, and provides the basis for the transferability of the experimental results to the human.

An additional focus of this study was the metastatic spread of the BL cells in the CAM. After dissection of the CAM, we were able to visualize BL2B95 cells in the stroma and abundantly in lymphatic vessels by immunofluorescence staining and TEM. BL2B95 cells are present in the lymphatics at great distances from the primary inoculation site. The BL2B95 cells that have migrated furthest are almost exclusively located in the lumen of lymphatics. This indicated that the cells had spread via the lymphatics to distant sites. To validate this observation, we applied an intravital real-time imaging approach with BL2-GFP cells. BL2-GFP cells showed the same migration pattern in the CAM as the BL2B95 cells. Single BL2-GFP cells could be seen leaving the tumor as early as 2 days after inoculation, providing evidence for early micrometastasis formation. The cells thereby migrated along the outside of CAM blood vessels, which is the typical localization of CAM lymphatics. Only one or two days later, the formation of metastases can be observed at distant sites. In numerous specimens, no significant numbers of tumor cells were present between the metastatic foci and the primary tumors, strongly indicating that the distant foci are the result of lymphogenic spread. Nevertheless, migration of BL2-GFP cells in the ECM adjacent to the primary tumor occurred as well, but the cells migrated only shorter distances. It may be of interest to study if the travelling of cells within CAM lymphatics is restricted to malignant cells or if normal human leukocytes disseminate via chicken lymphatics as well. If so, the CAM might be a suitable model for studies on B-cell homing.

In summary, our data show that the CAM is an excellent model to study tumor-stroma interactions with a focus on tumor angiogenesis, lymphangiogenesis and metastasis formation. Our results are in concord with cancer studies employing the CAM and expand the characterization of the CAM model to hematological malignancies [9,10,13,15].

## Conclusions

Our data show that the CAM is an excellent *in vivo* model for NHL research, but has until recently been underestimated with regards to its similarities with primary human lymphoma. We would like to stress that, depending on the scientific questions, the CAM model may well be used instead of mouse models in preclinical studies. In addition to the pros illustrated above, further advantages of the CAM are the low costs per animal, the convenient handling and the minor bureaucratic effort (summarized in Table 2). For the testing of new drugs, the CAM model can be interposed between cell culture and mouse experiments to serve as an *in vivo* screening platform [13,15], as we have recently shown for the anti-tumor drug imipramine-blue [10]. This might save scientific resources and accelerate the development of new chemotherapeutics due to the higher throughput and the earlier focus on promising drugs, especially those that may alter their effectiveness due to tumor-stroma interactions. Additionally, real-time imaging approaches of cells migrating within the lymphatics can be used to study the mechanisms of lymphogenic metastasis, and probably B-cell homing.

**Table 2:** Characteristics of BL tumors in chick and mouse xenograft models compared to the human disease

**Feature/host**	**Chick (CAM xenograft)**	**Mouse ((NOD-) SCID xenograft)**	**Human**
***Characteristics of BL tumors***			
Formation of solid tumors	+[9,17,26]	+[27,28]	+[3]
Lymphogenic dissemination	+[9] (Figures 3, 4, 5 and 6)	+[27]	+[29]
Distant organ metastasis	Not yet reported	+[27]	+[30]
Macrophage infiltration of tumor (‘starry sky’)	+ (Figures 1E, F; 3A)	+[23,31]	+[32]
Tumor cell morphology	-Blast-like	-Blast-like	- Blast-like
	-Basophilic cytoplasm	-Basophilic cytoplasm	- Basophilic cytoplasm
	-Lipid vacuoles	-Lipid vacuoles	-Lipid vacuoles
	-Prominent nucleoli (Figures 1, 2, 3, 4 and 5)	-Prominent nucleoli [33]	-Prominent nucleoli [34]
Lymphatic/blood vessel formation	+ [9] (Figure 4B, C)/+[18]	-/+[18]	+[35]/+[36]
Ki67^+^% of tumor cells	>90% (Figure 1E)	>90% [31]	90-100% [37]
***Experimental parameters***			
Immune status of host	Weakly immunocompetent (developing immune system)	Immunodeficient (depletion of B- and T-cells)	Mature immune system
Investigation period	Max. 8 -9 days	Weeks - months	Weeks - years
Bureaucratic efforts	None*	Medium (Animal experiment)	High (Clinical trial)
Premises	Incubator, Cell culture and imaging devices	Animal facility, Cell culture and imaging devices	Fully equipped hospital and staff
Costs/n	2 $	100 $[38]	−11000 $[39]

*According to German protection of animals act.

## Abbreviations

BECs: Blood endothelial cells; BL: Burkitt lymphoma; BL2-GFP: GFP-transfected BL2 cell line; BL2B95: Burkitt lymphoma cell line BL2, EBV serotype B95-8; CAM: Chorioallantoic membrane; CD: Cluster of differentiation; ECM: Extra-cellular matrix; Ki67: Antigen Ki67 (MKI67); LECs: Lymphatic endothelial cells; NHL: Non-Hodgkin lymphoma; TdT: Terminal desoxyribonucleotidyltransferase.

## Competing interests

The authors declare that they have no competing interests.

## Authors’ contributions

MK designed and performed experiments, and worked on the manuscript. DK and SE provided cell lines, designed experiments, and prepared the manuscript. JW and JB designed experiments, analyzed data, and prepared the manuscript. All authors read and approved the final manuscript.

## Pre-publication history

The pre-publication history for this paper can be accessed here:

http://www.biomedcentral.com/1471-2407/14/339/prepub

## Supplementary Material

Additional file 1: Figure S1 Characterization of BL2 cell line stably expressing GFP. **A**: Map of pGIPZ (Thermo Scientific) used for lentiviral transduction of BL2 to express a scrambled control shRNA (non-silencing control, ns-c) along with GFP. **B**: Flow cytometry illustrating GFP fluorescence in BL-2 ns-c* GFP cells (BL2-GFP) in comparison to the parental cell line BL-2. **C**: Immunoblot analysis of Bcl-6 and c-Myc in cell lysates showing that stable lentiviral transduction had no influence on protein levels of these transcription factors in two independently established GFP expressing BL-2 cell lines. Alpha-Tubulin served as loading control. **D**: Expression of control shRNA-GFP in two transfectants (BL-2 ns-c GFP and BL-2 ns-c* GFP) did not alter cell proliferation according to ^3^H thymidine assay. Shown is the relative thymidine uptake within 16 h. The level in BL-2 was set to 1. Click here for file

Additional file 2: Figure S2 Exo ovo chick chorioallantoic membrane assay. **A-G**: Pictures show chick embryos grown outside of the eggshell (*ex ovo*). The incubation day is indicated in the lower right corner. Insert in **A** shows the extension of the allantois. **H**: Chicken embryo in a weighing boat placed in a cell culture flask with a reclosable lid. **I**: Tumor cell inoculation on a d11 embryo. Arrow shows the site of tumor cell engraftment (50% Matrigel) and dotted lines show sites of direct cell applications (0% Matrigel, 10^6^ cells in 10 μL BL medium). Click here for file

Additional file 3: Figure S3 Intravital imaging of BL2-GFP cells in the *ex ovo* CAM model. **A-D”**: Time lapse images of BL2-GFP cells, which were grafted in 50% Matrigel on the CAM. BL2-GFP cell show a bright green fluorescence, CAM tissue shows a weak greenish autofluorescence, and blood vessels appear black. Pictures were taken every 24 h, beginning 48 h post inoculation (p.i.). The magnification of the pictures is indicated. The yellow rectangles in **A**, **A’** and **B** mark regions shown at higher magnification in **B-D**. **E-G** show distant micrometastases of the specimen shown in **A**. Click here for file

Additional file 4: Figure S4 Immunofluorescence staining of CAM. **A**: Prox1 stains nuclei of lymphatic endothelial cells. **B**: DAPI staining shows blood vessels, due to the nucleated chick erythrocytes. **C**: Merged picture illustrates the close proximity of lymphatics and blood vessels. Larger blood vessels are flanked by lymphatic collectors. Click here for file
