# Supplementary figures and images for: The chick chorioallantoic membrane as an in vivo xenograft model for Burkitt lymphoma
Source: BMC Cancer. 2014 May 18;14:339. doi: 10.1186/1471-2407-14-339 (PMC4036709; doi:10.1186/1471-2407-14-339)

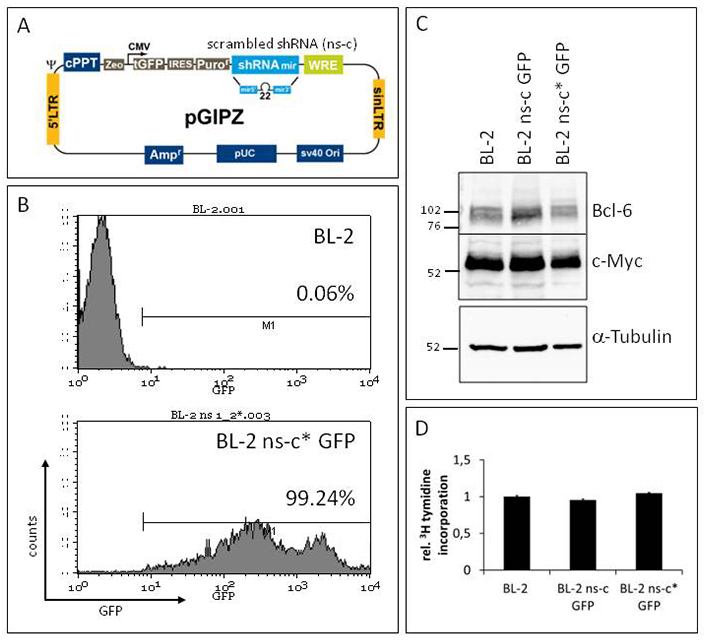

Supplement: Additional file 1: Figure S1 — Characterization of BL2 cell line stably expressing GFP. A: Map of pGIPZ (Thermo Scientific) used for lentiviral transduction of BL2 to express a scrambled control shRNA (non-silencing control, ns-c) along with GFP. B: Flow cytometry illustrating GFP fluorescence in BL-2 ns-c* GFP cells (BL2-GFP) in comparison to the parental cell line BL-2. C: Immunoblot analysis of Bcl-6 and c-Myc in cell lysates showing that stable lentiviral transduction had no influence on protein levels of these transcription factors in two independently established GFP expressing BL-2 cell lines. Alpha-Tubulin served as loading control. D: Expression of control shRNA-GFP in two transfectants (BL-2 ns-c GFP and BL-2 ns-c* GFP) did not alter cell proliferation according to 3H thymidine assay. Shown is the relative thymidine uptake within 16 h. The level in BL-2 was set to 1. [file 1471-2407-14-339-S1.jpeg]

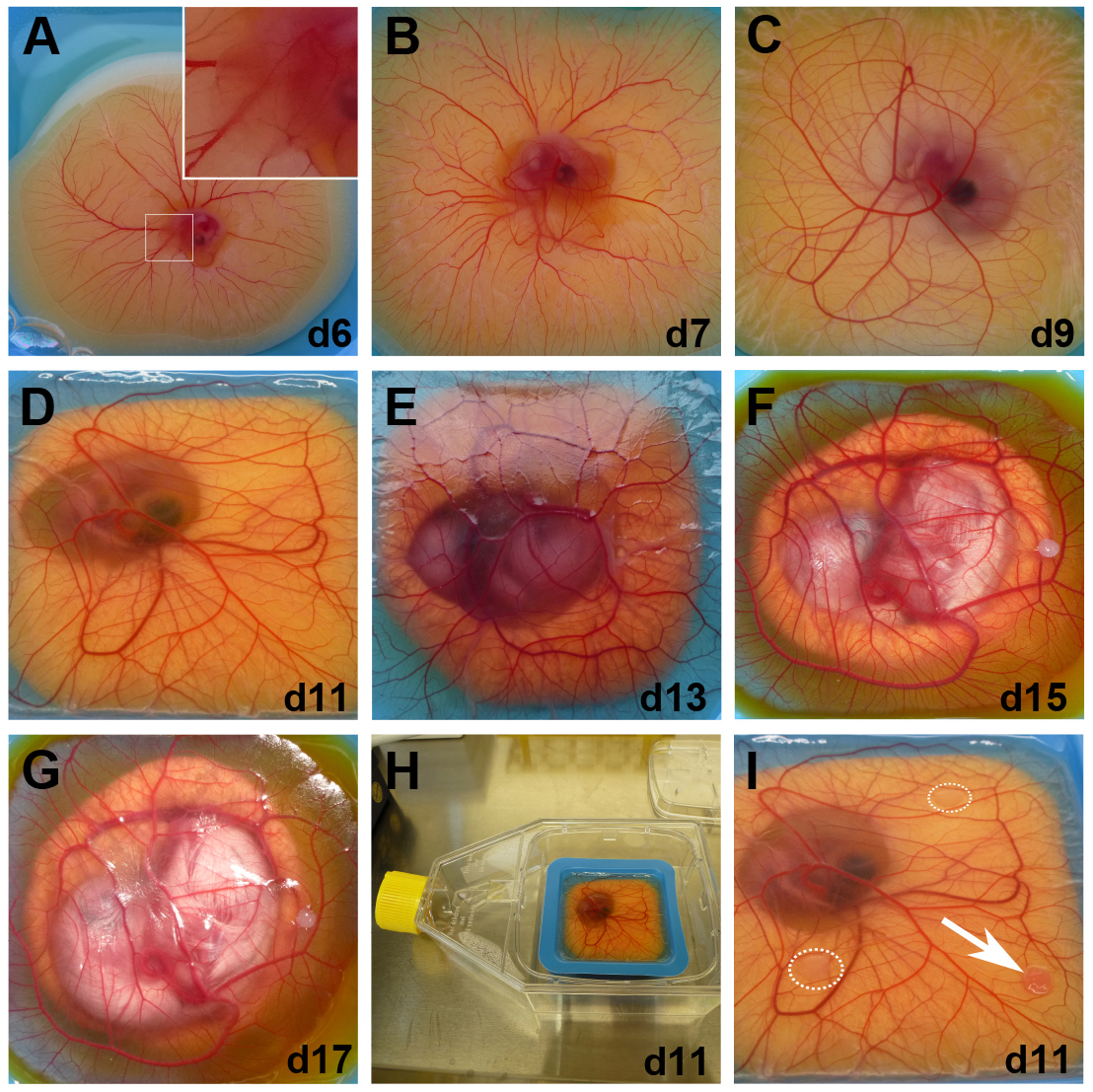

Supplement: Additional file 2: Figure S2 — Exo ovo chick chorioallantoic membrane assay. A-G: Pictures show chick embryos grown outside of the eggshell (ex ovo). The incubation day is indicated in the lower right corner. Insert in A shows the extension of the allantois. H: Chicken embryo in a weighing boat placed in a cell culture flask with a reclosable lid. I: Tumor cell inoculation on a d11 embryo. Arrow shows the site of tumor cell engraftment (50% Matrigel) and dotted lines show sites of direct cell applications (0% Matrigel, 106 cells in 10 μL BL medium). [file 1471-2407-14-339-S2.jpeg]

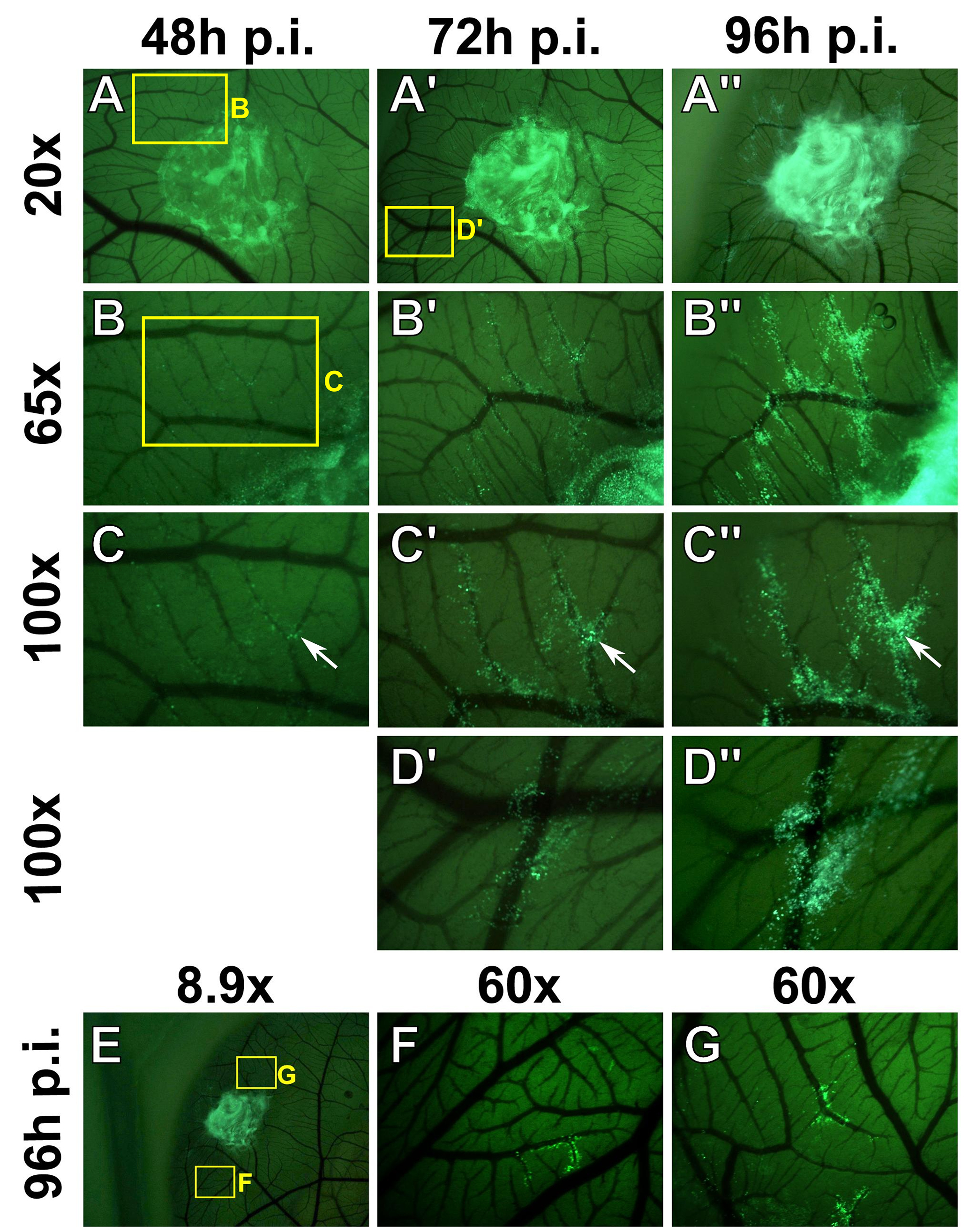

Supplement: Additional file 3: Figure S3 — Intravital imaging of BL2-GFP cells in the ex ovo CAM model. A-D”: Time lapse images of BL2-GFP cells, which were grafted in 50% Matrigel on the CAM. BL2-GFP cell show a bright green fluorescence, CAM tissue shows a weak greenish autofluorescence, and blood vessels appear black. Pictures were taken every 24 h, beginning 48 h post inoculation (p.i.). The magnification of the pictures is indicated. The yellow rectangles in A, A’ and B mark regions shown at higher magnification in B-D. E-G show distant micrometastases of the specimen shown in A. [file 1471-2407-14-339-S3.jpeg]

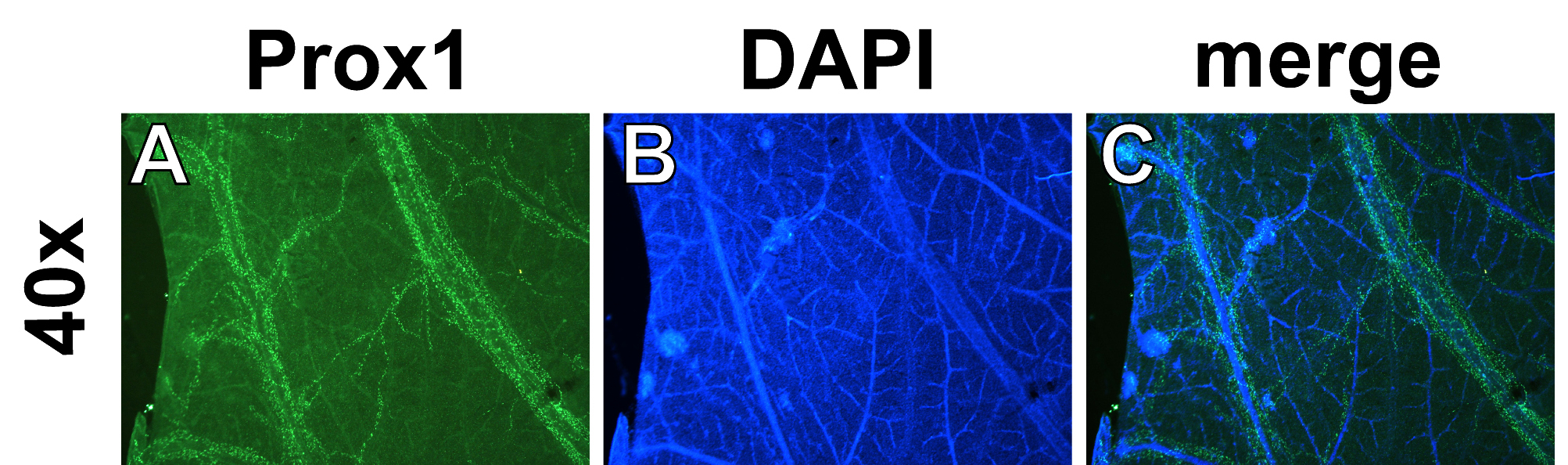

Supplement: Additional file 4: Figure S4 — Immunofluorescence staining of CAM. A: Prox1 stains nuclei of lymphatic endothelial cells. B: DAPI staining shows blood vessels, due to the nucleated chick erythrocytes. C: Merged picture illustrates the close proximity of lymphatics and blood vessels. Larger blood vessels are flanked by lymphatic collectors. [file 1471-2407-14-339-S4.jpeg]
